# Efficacy of Orthognathic Surgery in OSAS Patients: A Systematic Review and Meta‐Analysis

**DOI:** 10.1111/joor.13936

**Published:** 2025-01-24

**Authors:** Syed Akbar Ali, Maria Maddalena Marrapodi, Ganiga Channaiah Shivakumar, Sahana Shivakumar, Jyothikiran Hurkadle, Marco Cicciù, Giuseppe Minervini

**Affiliations:** ^1^ Department of Orthodontics & Dentofacial Orthopaedics People's College of Dental Sciences & Research Centre People's University Bhopal India; ^2^ Department of Woman, Child and General and Specialist Surgery University of Campania “Luigi Vanvitelli” Naples Italy; ^3^ Department of Oral Medicine and Radiology, People's College of Dental Sciences and Research Centre People's University Bhopal India; ^4^ Department of Public Health Dentistry People's College of Dental Sciences and Research Centre People's University Bhopal India; ^5^ Department of Orthodontics and Dentofacial Orthopaedics JSS Dental College and Hospital, JSS Academy of Higher Education and Research Mysuru India; ^6^ Department of Biomedical and Surgical and Biomedical Sciences Catania University Catania Italy; ^7^ Saveetha Dental College and Hospitals, Saveetha Institute of Medical and Technical Sciences (SIMATS) Saveetha University Chennai India; ^8^ Multidisciplinary Department of Medical‐Surgical and Odontostomatological Specialties University of Campania “Luigi Vanvitelli” Naples Italy

**Keywords:** apnea‐hypopnea index, Epworth sleepiness scale, meta‐analysis, obstructive sleep apnea syndrome, orthognathic surgery, systematic review, treatment efficacy

## Abstract

**Background:**

Obstructive sleep apnea syndrome (OSAS) is a prevalent condition characterised by repeated episodes of partial or complete obstruction of the upper airway during sleep, leading to disrupted sleep and associated morbidities. Orthognathic surgery (OGS) has been proposed as a treatment option for OSAS, aimed at anatomically repositioning the maxillofacial structures to alleviate airway obstruction. This systematic review and meta‐analysis aimed to evaluate the efficacy of OGS in reducing apnea‐hypopnea index (AHI) and Epworth Sleepiness Scale (ESS) scores among OSAS patients.

**Methods:**

We conducted a comprehensive literature search across multiple databases for studies assessing the outcomes of OGS in OSAS patients, focusing on changes in AHI and ESS scores. The inclusion criteria encompassed observational studies, cohort studies, and randomised control trials. Data extraction and quality assessment were performed independently by two reviewers. Random‐effects meta‐analysis was utilised to pool mean differences (MD) of AHI and ESS scores preoperatively and postoperatively, with 95% confidence intervals (CI) calculated.

**Results:**

A total of 8 studies met the inclusion criteria, where OGS was shown to be slightly more effective in correcting OSAS than the other modalities assessed, primarily CPAP. The pooled MD for AHI demonstrated a significant reduction in scores post‐OGS (MD = 29.84, 95% CI: 14.17–45.50, *p* < 0.0001) with substantial heterogeneity (*I* ^2^ = 95%). For ESS, the pooled MD indicated a non‐significant reduction (MD = 1.91, 95% CI: −1.29 to 5.12, *p* = 0.24) with high heterogeneity (*I* ^2^ = 81%).

**Conclusion:**

Orthognathic surgery appears to be an effective intervention for reducing AHI in patients with OSAS, suggesting a potential to improve the objective measures of sleep apnea. However, the effect on subjective sleepiness scores, as evaluated by ESS, was not statistically significant. The high heterogeneity among studies warrants individualised patient assessment when considering OGS for OSAS. Further research is needed to identify factors contributing to the variability of outcomes and to assess the long‐term benefits and risks associated with the procedure.

## Graphical abstract

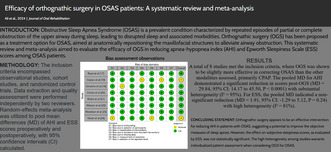

## 1 Introduction

Prevalent and significantly burdening public health, obstructive sleep apnea syndrome (OSAS) is a disorder characterised by repetitive episodes of partial or complete upper airway obstruction during sleep [1]. This condition can induce fragmented sleep and daytime sleepiness while also potentially causing myriad cardiovascular and metabolic complications [2, 3]. Several treatment modalities exist; however, managing OSAS presents significant challenges primarily because patients exhibit variable treatment responses and adherence levels.

Increasing recognition of orthognathic surgery (OGS), a collective term encompassing various surgical procedures on the jaw to correct structural and growth‐related conditions as well as sleep apnea among others, emerges as a potential therapeutic option for OSAS [4]. These procedures seek to ameliorate airflow obstruction by modifying anatomical structures in the upper airway; thus mitigating symptoms and complications of OSAS [5]. The ongoing debate subjects the efficacy of OGS in treating OSAS: some studies report significant improvements in OSAS symptoms and quality of life post‐operation, while others present less promising results [6]. Further, limited and inconclusive existing reviews on this topic often suffer from two primary obstacles—the heterogeneity among studies; a lack of robust, high‐quality evidence [7].

Maxillomandibular advancement (MMA) is a surgical intervention that has demonstrated efficacy in the treatment of obstructive sleep apnea (OSA), with success rates hovering around 80%, consistently observed in both short‐term and long‐term follow‐ups [8]. While the surgical procedure often leads to cosmetic enhancements, particularly in the facial profile, there can be instances where the aesthetic outcomes may not be favourable, particularly around the paranasal area [9].

To address the challenge of augmenting structurally deficient areas without disrupting the aesthetic harmony of unaffected facial regions, differential advancement techniques are employed [10]. Specifically, when the advancement requirements of the mandible do not align with those of the maxilla, a strategic approach known as counterclockwise rotation of the occlusal plane (CCWROP) is employed [11]. This surgical technique is instrumental in advancing the anterior mandibular region, notably the pogonion, thereby achieving both the cosmetic objectives and the primary therapeutic goals for OSA management, which is the expansion of the pharyngeal airspace [12, 13, 14]. This nuanced approach to MMA surgery allows for a tailored alteration of facial structure, directly contributing to the amelioration of OSA symptoms while concurrently respecting the patient's aesthetic integrity.

Considering these factors, this systematic review and meta‐analysis set out to comprehensively evaluate the efficacy of OGS in managing OSAS. We aim to robustly quantify the treatment effect by consolidating data from pertinent studies, while also probing potential sources of heterogeneity. Our aspiration is that our findings will significantly enrich existing knowledge, shape upcoming research directions; moreover, they should serve as a crucial reference for making informed clinical decisions regarding OSAS management.

## 2 Materials and Methods

### 2.1 Review Design

**FIGURE 1:**
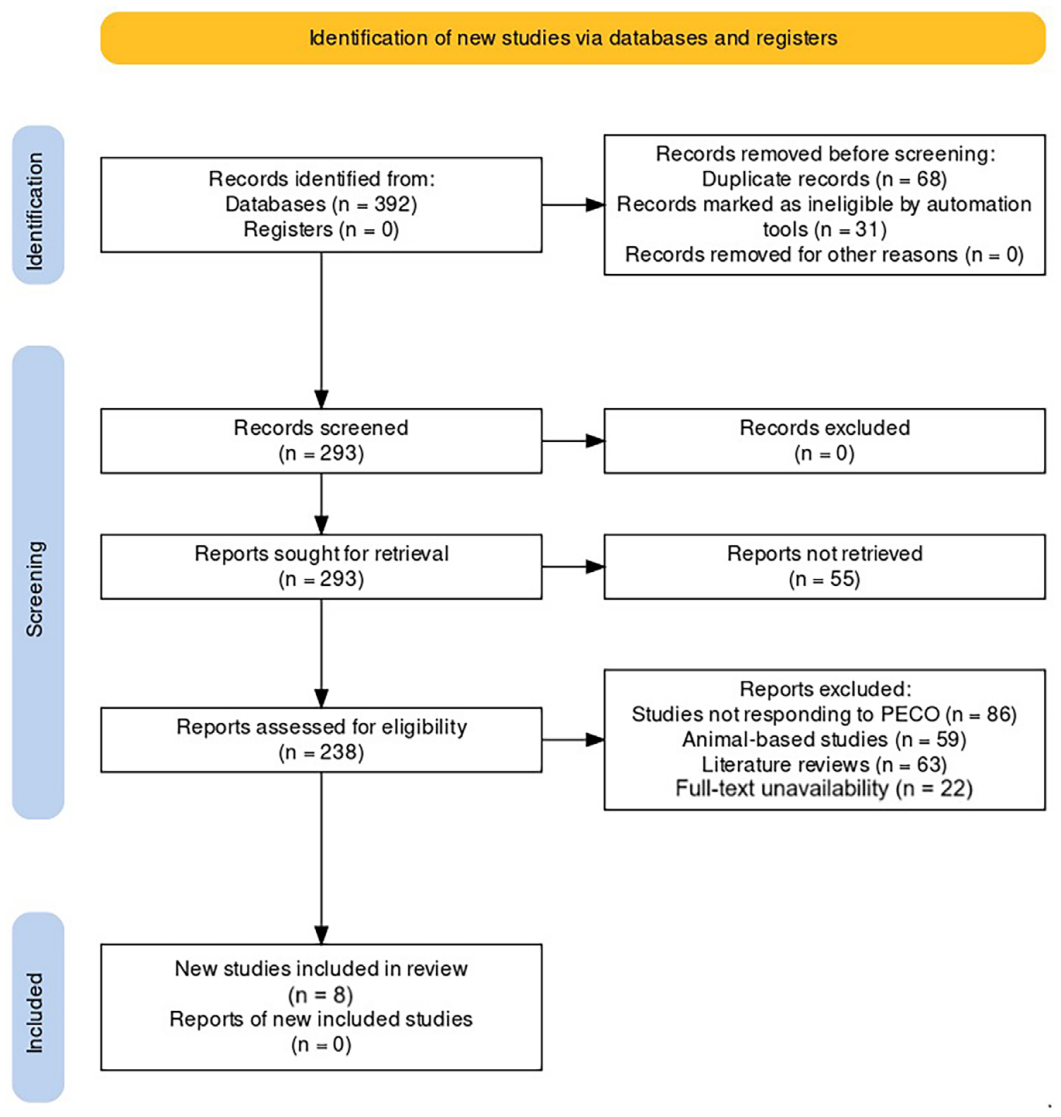
PRISMA protocol representation for the review.

The PRISMA guidelines [15] were utilised for reporting this review, with Figure 1 showing the results of the article selection process.

The PECO framework for this review is given as follows:

- *Population* (*P*): The review focused on patients diagnosed with OSAS. The inclusion criteria encompassed all age groups, genders, and the severity of OSAS, as diagnosed by standard polysomnography or equivalent sleep studies.
- *Exposure* (*E*): The exposure of interest was OGS, including various surgical techniques such as MMA, genioglossus advancement, and hyoid myotomy and suspension, among others. The review included studies that detailed pre‐surgical and post‐surgical evaluations.
- *Comparator* (*C*): The comparators included non‐surgical treatment methods such as continuous positive airway pressure (CPAP), mandibular advancement devices (MAD), and lifestyle interventions, as well as studies that reported on the natural progression of OSAS without intervention.
- *Outcome* (*O*): The primary outcomes measured were the post‐surgical apnea‐hypopnea index (AHI) and the Epworth Sleepiness Scale (ESS) score. Secondary outcomes included quality of life assessments, surgical morbidity and other polysomnographic parameters.

### 2.2 Search Protocol

For the database search protocol, eight electronic databases were comprehensively searched using a combination of Boolean operators and Medical Subject Headings (MeSH) keywords. The databases included PubMed, EMBASE, Scopus, Web of Science, Cochrane Library, CINAHL, PsycINFO, and Google Scholar. The search strategy was constructed using terms for "orthognathic surgery," "maxillomandibular advancement," "obstructive sleep apnea," and "sleep apnea syndromes," combined with MeSH terms such as "sleep apnea, obstructive" and "mandibular advancement." Boolean operators "AND" and "OR" were employed to refine the search. Table 1 shows the descriptive search strings that were employed across the different databases.

**TABLE 1:** Search strings utilised across the studies.

Database	Search string
PubMed	("Orthognathic Surgery"[Mesh] OR "Maxillomandibular Advancement"[Mesh]) AND ("Sleep Apnea Syndromes"[Mesh] OR "Obstructive Sleep Apnea"[Mesh])
EMBASE	('orthognathic surgery'/exp OR 'maxillomandibular advancement'/exp) AND ('sleep apnea syndrome'/exp OR 'obstructive sleep apnea'/exp)
Scopus	(TITLE‐ABS‐KEY (orthognathic AND surgery) OR TITLE‐ABS‐KEY (maxillomandibular AND advancement)) AND (TITLE‐ABS‐KEY (obstructive AND "sleep apnea"))
Web of Science	(TS=(orthognathic surgery) OR TS=(maxillomandibular advancement)) AND (TS=(obstructive sleep apnea syndrome) OR TS=(OSAS))
Cochrane Library	(MeSH descriptor: [Orthognathic Surgical Procedures] OR MeSH descriptor: [Maxillomandibular Advancement]) AND (MeSH descriptor: [Sleep Apnea, Obstructive])
CINAHL	(MH "Orthognathic Surgery" OR MH "Maxillomandibular Advancement") AND (MH "Sleep Apnea Syndromes" OR MH "Obstructive Sleep Apnea")
PsycINFO	(DE "Orthognathic Surgery" OR DE "Maxillomandibular Advancement") AND (DE "Sleep Apnea Syndromes" OR DE "Obstructive Sleep Apnea")
Google Scholar	allintitle: orthognathic surgery OR maxillomandibular advancement AND obstructive sleep apnea

### 2.3 Inclusion and Exclusion Criterion

Table 2 shows the selection criterion that were utilised for this review.

**TABLE 2:** Inclusion and exclusion criteria utilised for the review.

Criteria type	Description
Inclusion criteria	Studies involving patients diagnosed with OSAS Studies evaluating the efficacy of OGS interventions Outcomes measured included post‐surgical AHI, ESS score, and other relevant polysomnographic parameters Both randomised controlled trials (RCTs) and observational studies (cohort, case–control, and cross‐sectional studies) Studies that reported sufficient data to calculate effect sizes for meta‐analysis
Exclusion criteria	Studies not focusing on orthognathic surgery as an intervention for OSAS Case reports, letters, editorials, reviews, and animal studies Articles not published in English Studies without clear outcome measures or insufficient data for inclusion in the meta‐analysis Studies where OGS was combined with other surgical procedures, and the effects could not be isolated Studies with populations having syndromic conditions or craniofacial anomalies that could confound the outcomes of orthognathic surgery Duplicates or studies with overlapping datasets

### 2.4 Data Extraction Protocol

A standardised data extraction form was developed and pilot‐tested on a small sample of included studies to ensure its comprehensiveness and functionality. The form included fields for study identification (author, year of publication, country), study design (randomised controlled trial, cohort study, etc.), participant demographics (age, sex, BMI, severity of OSAS), details of the orthognathic surgical procedure(s), comparator interventions and outcome measures (post‐surgical AHI, ESS score and other relevant polysomnographic parameters). The data extraction process was conducted independently by two reviewers, M.M. and G.C.S. In cases where there was a disagreement between them, S.A.A. acted as the third reviewer to provide a final decision. To quantify the agreement between reviewers, an interrater reliability test was conducted using Cohen's Kappa coefficient. The Kappa values obtained ranged from 0.80 to 0.92 across different data categories, indicating a substantial to almost perfect agreement. These values reflected a high level of consistency between the reviewers in the data extraction process.

Additionally, for continuous variables, means, standard deviations and sample sizes were extracted, while for dichotomous outcomes, the number of events and the total number of participants were recorded. Where data were incomplete or unclear, the corresponding authors of the studies were contacted for clarification.

### 2.5 Bias Assessment Strategy

**FIGURE 2:**
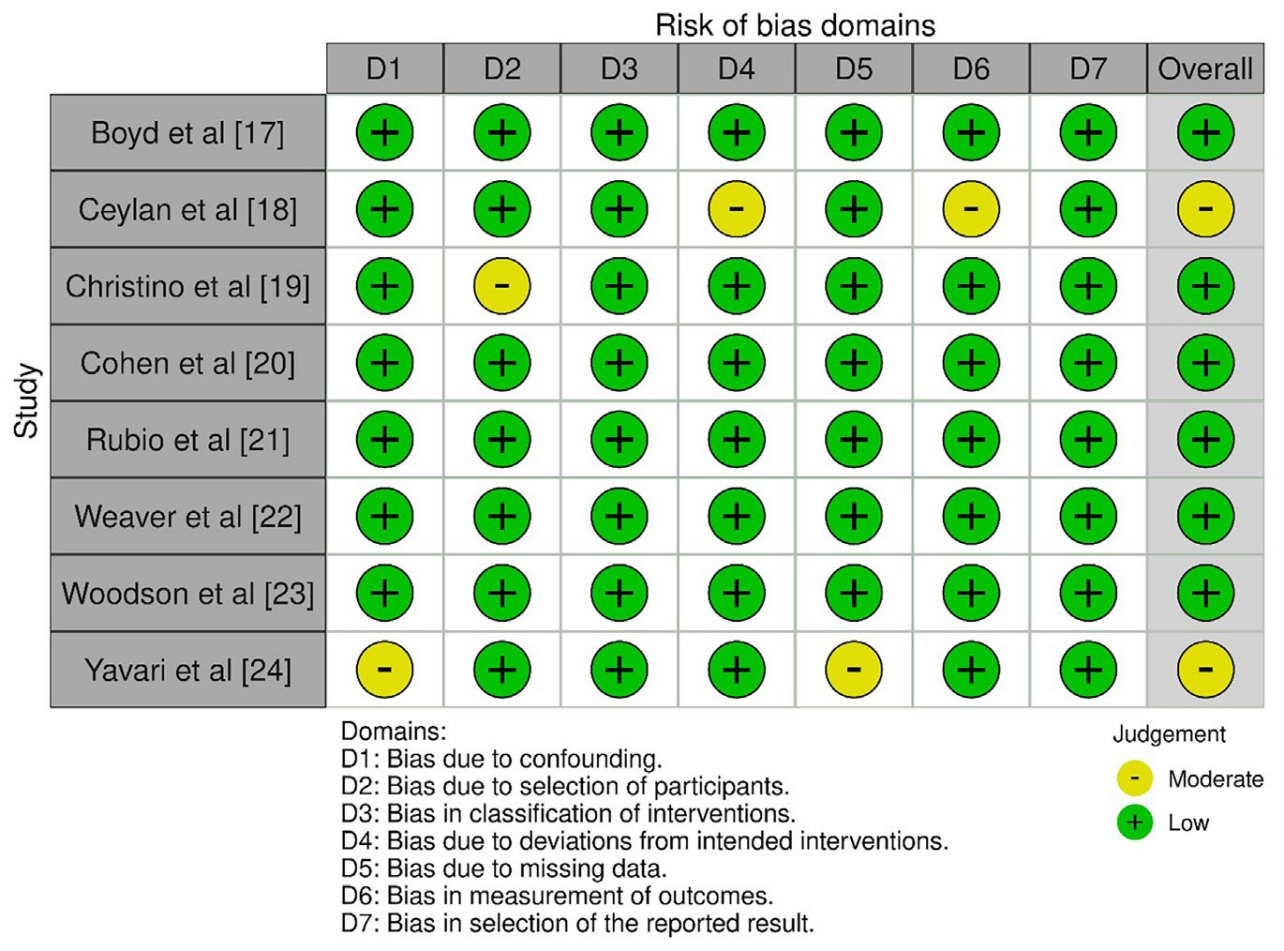
Bias representation across different domains of the studies included in the review.

The bias assessment protocol was meticulously developed to evaluate the risk of bias in the included non‐randomised studies of interventions. The chosen instrument for this purpose was the ROBINS‐I tool (Risk Of Bias In Non‐randomised Studies—of Interventions) [16], the results of which have been shown in Figure 2 and Table 3.

**TABLE 3:** Studies included in the review and their observed assessments.

Study ID	Objective	Patient details	Methods/Interventions	Principal findings	Success rate
Boyd et al. [17]	To determine the long‐term clinical effectiveness and safety of MMA for the treatment of moderate to severe OSA	30 adult patients (80% men, mean age 50.5 ± 9.6 years) who underwent MMA > 2 years ago	Prospective two‐center cohort study with primary outcome measure as AHI and secondary measures including BP, ESS, and FOSQ	Significant decreases in AHI (mean of 49–10.9 events/h), with 46.7% achieving AHI < 5 and 83.4% achieving AHI ≤ 15 events/h. Mean diastolic BP, ESS improved, and FOSQ increased. Few long‐term treatment‐related adverse events. Suggests MMA is effective and safe long‐term treatment for moderate‐to‐severe OSA	83.4% (AHI ≤ 15 events/h)
Ceylan et al. [18]	To compare the efficacy of TCRFTVR for the soft palate and base of the tongue with that of nasal CPAP in primary treatment of mild to moderate OSA	47 patients with mild to moderate OSA treated between Jan 1, 2003, and Oct 31, 2006	26 patients underwent TCRFTVR and 21 underwent nasal CPAP	Both TCRFTVR and CPAP showed meaningful improvements in ESS and polysomnography variables at 12 months compared with baseline. No significant difference between the groups posttreatment	TCRFTVR: 53.8%; CPAP: 52.4%
Christino et al. [19]	To evaluate the impact of CCWROP on pharynx morphology and polysomnography in MMA surgery to treat OSA patients	38 OSA patients treated by MMA, classified into two groups: with CCWROP (R, *n* = 19) and without CCWROP (NR, *n* = 19)	MMA surgery with (R) and without (NR) CCWROP	R showed an 80% reduction in AHI, compared to a 62% reduction in NR. Significant improvements in total, retropalatal, and retrolingual volumes and minimum axial area, especially in the retrolingual region. Anterior mandibular advancement of 0.71 mm per degree of CCWROP significantly influenced outcomes, with R showing better results than NR in all assessed parameters	Not specified
Cohen‐Levy et al. [20]	To assess changes in adult male patients' profiles treated for OSAS with MMA and to measure patient perception of changes	15 male patients, mean age 42 (20–59), BMI 26.60 kg/m^2^ (22–29), AHI 50.9 (19–85)	MMA surgery, facial photography, lateral cephalography, polysomnography, self‐assessment questionnaire	MMA success rate for OSAS was 80% (AHI < 15). Profiles preferred by 85% of panels post‐op. Mean advancement: maxilla 8.4 mm, mandible 10.8 mm	80% (12/15)
Rubio‐Bueno et al. [21]	To investigate whether normalisation of the MOP is a determinant factor in curing OSA	34 subjects, mean age 41 ± 14, 58.8% female, ESS 17.4 ± 5.4, AHI 38.3 ± 10.7/h	MMA surgery, pre‐op and post‐op 3D scans, polysomnograms	Post‐op AHI 6.5 ± 4.3/h, 52.94% cured, MOP normalisation and mandibular advancement 6‐10 mm identified as best predictor variables	52.94% cured
Weaver et al. [22]	To test whether CPAP is associated with better survival than UPPP	Sleep apnea patients treated with CPAP or UPPP in Veteran Affairs facilities from 1997 to 2001	Retrospective cohort database study, Cox regression	UPPP conferred a survival advantage over CPAP after adjustment (31% higher probability of being dead with CPAP)	Not applicable
Woodson et al. [23]	To determine the effectiveness of TCRFTA or CPAP for the treatment of mild to moderate OSAS	90 adults with mild to moderate OSAS randomised into TCRFTA (*n* = 30), CPAP (*n* = 30), and sham‐placebo (*n* = 30) groups	Randomised, placebo‐controlled, 2‐site trial comparing TCRFTA and CPAP with sham‐placebo using intention‐to‐treat analysis	TCRFTA improved reaction time, QOL, and subjective sleepiness. Compared with sham‐placebo, TCRFTA improved QOL, airway volume, apnea index, and respiratory arousal index. CPAP improved QOL and sleepiness compared with baseline and QOL when compared with sham‐placebo. No significant differences were seen between TCRFTA and CPAP outcomes	Not explicitly stated
Yavari et al. [24]	To assess the impact of isolated mandibular setback surgery on the risk of OSA	30 patients (15 females, 15 males) with skeletal Class III deformity, mean age 25.77 ± 4.76	Double‐blinded prospective quasi‐experimental study with isolated mandibular setback surgery and SBQ assessments at T0, T1, and T2	SBQ score increased at T1 but decreased to near baseline at T2 (*p* < 0.001). Setback > 5 mm increased risk for OSA (*p* < 0.005). Setback < 5 mm did not significantly increase OSA risk	Not explicitly stated

### 2.6 Meta‐Analysis Protocol

The meta‐analysis protocol was developed to establish the efficacy of OGS with a focus on quantifying improvements across AHI and ESS scores. Prior to the commencement of the meta‐analysis, the protocol specified that all included studies would be pooled using a random‐effects (RE) model, due to the anticipated clinical and methodological heterogeneity across studies. This model assumes that the effect sizes from the studies are not identical but follow a distribution. The RE model was considered appropriate for the analysis as it accounts for variation both within and between the studies. The primary outcomes of interest, AHI and ESS scores, were continuous variables, and the measure of effect was expressed as the mean difference (MD) with 95% confidence intervals (CI). For each study, the mean post‐operative scores and standard deviations for AHI and ESS were used to calculate the MD between pre‐ and post‐operative values. Forest plots were generated for visual representation of the individual study effects and the pooled effect size. Each forest plot depicted the MD for each study with its corresponding 95% CI, along with the overall combined MD under the RE model. The weight of each study in the meta‐analysis was represented by the size of the square, which corresponded to the inverse variance of each study's effect estimate. The protocol included a thorough assessment of statistical heterogeneity using the *I* ^2^ statistic. *I* ^2^ values of 25%, 50% and 75% were indicative of low, moderate and high heterogeneity, respectively. In cases of high heterogeneity, sensitivity analyses were planned to explore potential sources, including methodological quality, sample size and variation in surgical technique.

## 3 Results

### 3.1 Patient Demographics Assessed

Boyd et al. [17] conducted research on 30 adult patients who had previously undergone MMA over 2 years prior. These patients, predominantly male with an average age of approximately 50 years, provided long‐term follow‐up data valuable for assessing the durability of MMA in treating OSA. Ceylan et al. [18] reviewed the treatment outcomes of 47 patients with mild to moderate OSA within a specific timeframe. The interventions compared transpalatal radiofrequency tissue volume reduction (TCRFTVR) for the soft palate and base of the tongue, as opposed to nasal CPAP, providing insight into less invasive treatment modalities. Christino et al. [19] focused their study on 38 OSA patients who also underwent MMA. These patients were divided into two cohorts based on whether the CCWROP was performed or not (NR). This distinction allowed for an analysis of the impact of CCWROP on the morphology of the pharynx and the outcomes of polysomnography, which is a critical tool in OSA assessment. Cohen et al. [20] limited their study to 15 male patients, providing a narrower but more controlled dataset. These patients were middle‐aged, with a mean body mass index (BMI) in the overweight range, and significant AHI scores indicating severe OSA. The scientific value of this study lies in its focus on the male demographic and the severe end of the OSA spectrum.

Rubio‐Bueno et al. [21] included 34 subjects of both genders, with a slight female majority, and a mean age in the early 40s. The Epworth Sleepiness Scale (ESS) and AHI were used to quantify symptom severity and frequency of apneas or hypopneas, thus addressing both the subjective experience of sleepiness and the objective measure of sleep disruption. Weaver et al. [22] looked at veteran patients treated with either CPAP or uvulopalatopharyngoplasty (UPPP) over a 4‐year period. The study's significance stems from its real‐world setting and the comparison of a medical device intervention with a surgical procedure. Woodson et al. [23] presented a randomised study involving 90 adults with mild to moderate OSA, comparing the outcomes of TCRFTA, CPAP, and a sham‐placebo group. Such a study design is pivotal for validating the efficacy of treatments by controlling for the placebo effect. Yavari et al. [24] involved 30 patients with a skeletal Class III deformity, evenly split by gender and with a mean age in the mid‐20s. This study provided data on a younger population and a specific craniofacial abnormality associated with OSA.

### 3.2 Interventions Assessed

Boyd et al. [17] conducted a prospective two‐centre cohort study focusing on MMA as a treatment for OSA. The primary outcome measure was AHI, with secondary measures including blood pressure (BP), ESS, and the Functional Outcomes of Sleep Questionnaire (FOSQ). These measures provided a comprehensive assessment of the physiological and quality‐of‐life outcomes post‐MMA. Ceylan et al. [18] compared TCRFTVR to CPAP in 47 patients diagnosed with mild to moderate OSA. This study's design allowed for an evaluation of the effectiveness of a less invasive radiofrequency technique against the more established CPAP therapy, using both subjective and objective sleep measures as endpoints. Christino et al. [19] researched the effects of MMA surgery with and without CCWROP in 38 OSA patients. This comparison provided insights into the impact of additional occlusal plane adjustment on the surgical outcome, which was assessed based on changes in AHI. Cohen‐Levy et al. [20] embraced a comprehensive approach by incorporating MMA surgery, facial photography, lateral cephalography, and polysomnography, complemented by self‐assessment questionnaires. This allowed for a multifaceted evaluation of surgery outcomes, encompassing both objective sleep parameters and patient‐perceived changes.

Rubio‐Bueno et al. [21] also examined the outcomes of MMA surgery, employing pre‐operative and post‐operative three‐dimensional scans and polysomnograms to assess changes in airway anatomy and sleep‐disordered breathing severity. This provided a detailed understanding of how structural changes post‐surgery correlated with functional sleep improvements. Weaver et al. [22] conducted a retrospective cohort database study and applied Cox regression analysis to evaluate treatment outcomes. The study likely focused on comparing the effectiveness of CPAP with UPPP over time in a Veteran Affairs patient population, providing valuable survival analysis data for these treatments. Woodson et al. [23] undertook a randomised, placebo‐controlled, two‐site trial comparing TCRFTA and CPAP with a sham‐placebo group. They employed an intention‐to‐treat analysis to ensure that the findings were representative of real‐world treatment adherence and effectiveness, thus providing robust evidence of treatment efficacy. Yavari et al. [24] conducted a double‐blinded prospective quasi‐experimental study examining the outcome of isolated mandibular setback surgery. Using the Sleep Breath Questionnaire (SBQ) assessed at three different time points (T0, T1 and T2), the study provided insight into the temporal changes in patient‐reported symptoms post‐surgery.

### 3.3 Principal Findings Assessed

Boyd et al. [17] found that MMA surgery led to a significant reduction in AHI, with a mean decrease from 49 to 10.9 events/h. A substantial portion of patients achieved an AHI of less than 5 events/h, and the majority reached an AHI of 15 events/h or less. Improvements in diastolic BP, ESS scores and FOSQ scores were also noted, supporting the efficacy and safety of MMA for OSA treatment. Ceylan et al. [18] showed that both TCRFTVR and CPAP resulted in improvements in OSA symptoms without significant differences between the two treatments, suggesting comparable effectiveness. Christino et al. [19] revealed that MMA surgery with CCWROP I led to a greater reduction in AHI and improvements in airway volumes compared to without CCWROP (NR), emphasising the benefits of additional occlusal plane rotation. Cohen et al. [20] reported a high success rate in reducing AHI post‐MMA surgery and a preference for postoperative facial profiles, with significant maxillary and mandibular advancements.

Rubio‐Bueno et al. [21] observed a significant reduction in postoperative AHI, with over half of the patients achieving an AHI of less than 5 events/h. Maxillofacial opening pattern normalisation and mandibular advancement were key predictors of treatment success. Weaver et al. [22] concluded that UPPP offered a survival advantage over CPAP, with a higher mortality risk associated with the latter after adjustments. Woodson et al. [23] determined that TCRFTA and CPAP both improved quality of life and sleepiness, with TCRFTA also improving airway volume and apnea index. However, there were no significant differences in outcomes between TCRFTA and CPAP. Yavari et al. [24] noted that isolated mandibular setback surgery increased the SBQ score initially, but it decreased to near baseline levels later on, with setbacks of more than 5 mm increasing OSA risk.

### 3.4 AHI Score Analysis

**FIGURE 3:**
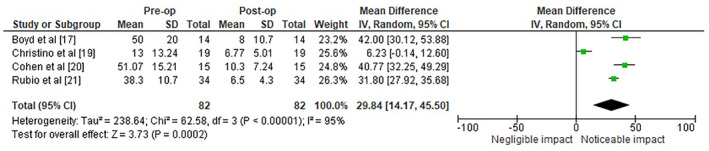
Efficacy of OGS for OSAS in terms of preoperative and postoperative AHI scores observed.

The forest plot in Figure 3 illustrates the MD in AHI scores before and after OGS. The total pooled MD across all studies was 29.84 events per hour, with a 95% CI of 14.17–45.50. This indicates a substantial overall reduction in AHI scores following OGS for OSAS. The heterogeneity of the data was high, with an *I* ^2^ statistic of 95% and a Tau^2^ of 238.64, which suggests that the studies' outcomes varied significantly. This high degree of heterogeneity is also reflected in the chi‐squared statistic of 62.58 with 3 degrees of freedom (*p* < 0.00001), confirming that the variability between studies is statistically significant. The test for overall effect yielded a *Z*‐score of 3.73, with a *p*‐value of 0.0002, indicating that the overall MD in AHI scores before and after OGS is statistically significant, and thus, OGS is effective in reducing the severity of OSAS.

### 3.5 ESS Score Analysis

**FIGURE 4:**
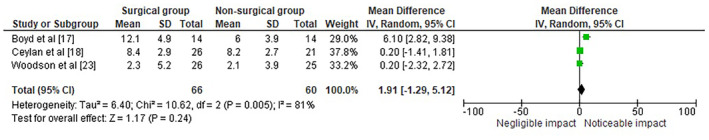
Efficacy of OGS for OSAS in terms of preoperative and postoperative ESS scores observed.

The forest plot in Figure 4 shows the MD in the ESS scores before and after OGS. The combined pooled MD for the ESS scores across all included studies was 1.91, with a 95% CI ranging from −1.29 to 5.12, indicating a small and not statistically significant reduction in sleepiness as measured by the ESS. This result is supported by a *Z*‐score of 1.17 and a corresponding *p*‐value of 0.24, indicating no significant change in sleepiness post‐surgery across the studies. Heterogeneity among the studies was significant, with an *I* ^2^ value of 81% and a Tau^2^ of 6.40. The chi‐squared statistic was 10.62 with 2 degrees of freedom (*p* = 0.005), indicating that the differences in outcomes between the studies are statistically significant and not likely due to chance.

## 4 Discussion

Boyd et al. [17] demonstrated the effectiveness of MMA in reducing the AHI, as well as improvements in BP, the ESS and the FOSQ. This finding was consistent with that of Rubio‐Bueno et al. [21], who also reported significant AHI reductions post‐MMA surgery. The congruity of these results underscores MMA's role as a robust surgical intervention for OSA. In contrast, Ceylan et al. [18] and Woodson et al. [23] explored the comparative effectiveness of TCRFTVR and CPAP. Both studies found these treatments to be comparably efficacious in symptom improvement, challenging the notion that CPAP is the sole gold standard for OSA management and supporting the viability of TCRFTVR as an alternative, particularly for patients with mild to moderate OSA.

Christino et al. [19] expanded upon the surgical approach by comparing the outcomes of MMA with and without CCWROP, finding that the inclusion of CCWROP yields greater reductions in AHI and increases in airway volume. This study provides a nuanced understanding that specific surgical enhancements can further optimise patient outcomes. Cohen et al. [20] offered a comprehensive evaluation of MMA surgery outcomes, including patient satisfaction with postoperative facial aesthetics, which was a novel aspect not specifically addressed in other studies. The positive reception of facial changes post‐MMA surgery aligns with the physical improvements reported by Boyd et al. [17] and Rubio‐Bueno et al. [21], yet it introduces an additional dimension to consider—patient satisfaction and the subjective valuation of aesthetic outcomes.

Weaver et al. [22] presented a unique perspective by employing survival analysis to compare UPPP with CPAP treatment, revealing a survival advantage for UPPP. This finding is noteworthy as it diverges from the other studies, which did not focus on long‐term survival but rather on symptomatic and functional outcomes. Yavari et al. [24] provided a cautionary insight into isolated mandibular setback surgery, indicating an initial increase in OSA risk followed by a return to near baseline levels, particularly with setbacks greater than 5 mm. This finding stands in stark contrast to the otherwise positive outcomes associated with MMA surgery and highlights the complexity of surgical interventions for OSA.

In the realm of therapeutic interventions for OSAS, MMA and CPAP therapies have been the focus of comparative efficacy investigations. A notable randomised clinical study [25] assessed these interventions over a 1‐year period, utilising at‐home level 3 unattended polysomnography for patient evaluation. The study delineated a substantial amelioration in AHI and ESS subsequent to both MMA and auto‐titrating positive airway pressure (APAP) therapies, with post‐intervention AHI averages descending to 8.1 from a pre‐MMA AHI of 56.8 and to 6.3 from a pre‐APAP AHI of 50.3. Corresponding ESS averages exhibited declines to 7.7 from a pre‐MMA ESS of 11.6 and to 5.9 from a pre‐APAP ESS of 11.2, indicating no significant discrepancy in efficacy between the two modalities.

Furthermore, MADs represent an alternative therapeutic option, particularly efficacious for patients with mild OSA. However, their success in treating moderate to severe OSA has not been as conclusive [26, 27]. A retrospective analysis comparing MMA to MADs in moderate‐to‐severe OSA patients revealed a marked superiority of MMA, with an odds ratio of 3.22 favouring MMA's efficacy in achieving a desirable therapeutic outcome, defined as an AHI reduction to fewer than 15 events per hour and a 50% decrease from baseline AHI [26].

In terms of adherence‐dependent therapies, both MADs and CPAP require ongoing commitment to use for sustained clinical effectiveness, with the necessity of periodic replacement of devices and associated components [28]. Long‐term outcomes post‐MMA, however, have been reported to yield significant and enduring reductions in subjective sleepiness, with Boyd et al. [17] noting that 90% of post‐MMA patients achieved normal ESS scores (≤ 10), with an average long‐term decrement of 5.6 points on the ESS.

These long‐term outcomes are in alignment with findings from a recent meta‐analysis suggesting that extensive CPAP utilisation (in excess of 4 h per night) correlates with a reduction in ESS scores by an average of 4.2 points [29, 30]. Additional studies corroborate these findings, with Antic et al. reporting ESS normalisation in 80.6% of patients with moderate to severe OSA following more than 7 h of nightly CPAP use. Patel et al.'s meta‐analysis also supports this trend, with severe OSA patients experiencing an average ESS score reduction of 4.75 points [30]. Moreover, the permanence of MMA's beneficial effects on sleepiness has been documented in both short‐term and long‐term follow‐ups, with studies evidencing 100% and 90% rates of ESS normalisation. This data collectively reinforce the enduring nature of MMA's impact on mitigating subjective sleepiness in the context of OSA treatment [31, 32].

Where UPPP was historically considered the cornerstone of surgical approaches to OSA, this review demonstrates how the outcomes, especially long‐term, might not be as friendly as other advanced procedures such as BRP or ESP [33, 34, 35, 36]. These newer techniques promise similar or better efficacy with fewer complications and faster recovery times. The potential adverse effects of UPPP include nasal regurgitation, speech impairment and prolonged postoperative pain, and its dependence on careful patient selection makes the search for better surgical alternative options for patients with OSA more meaningful.

Cammaroto et al. [33] highlighted that UPPP, as a procedure, although accepted and practiced, seems to provide less long‐term outcomes when compared to newer procedures such as BRP or ESP in the setting of multilevel surgery. The data obtained showed that BRP and ESP resulted in better postoperative AHI values and overall surgical success when compared with UPPP. Other significant differences in the studies were that UPPP was associated with prolonged recovery times and higher complication rates. This implies that though an improvement might be noticed immediately, in the long run, this does not appear to be a durable solution‐perhaps compared with newer techniques that aim to minimise complications and maximise efficacy.

Maniaci et al. [34] also compared UPPP with LP and found that although both surgeries had a significant improvement in the outcomes of patients, results with LP were very slightly better in AHI and levels of oxygen saturation. However, the differences were not significant suggesting that although the UPPP is an effective procedure, there exists alternative procedures that can have comparable or even better outcomes with fewer complications. This holds specially true for long‐term care of patients, as UPPP can sometimes cause long‐term side effects like dry throat, dysphagia and changes in speech.

Yousuf et al. [35] showed a bright view of UPPP with high success rates for appropriately selected patients based on clinical parameters like neck circumference and the Friedman staging system. However, they also mentioned that success is dependent on proper patient selection, which limits the wide applicability of UPPP as a panacea for all OSA patients. The dependence on strict patient criteria shows that UPPP is not suited to everyone thereby underlining the importance of proper preoperative assessment in avoiding postoperative complications.

According to Cincik et al. [36], postoperative pain is considerably higher in UPPP than in other procedures: laser‐assisted uvulopalatoplasty (LAUP), cautery‐assisted uvulopalatoplasty (CAUP). Although the three techniques were equal to each other in terms of the effectiveness of snoring reduction, the pain scores were among the highest for UPPP and recovery times were the longest. Moreover, complications such as velopharyngeal insufficiency, as well as secondary infections, are more often associated with UPPP. This reveals that although UPPP remains an option, particularly when newer techniques are not available, it brings with it considerable disadvantages that may worsen long‐term recovery and be less satisfying to the patient.

### 4.1 Limitations of This Review

The limitations of the studies in question are multifaceted and crucial for a comprehensive understanding of the findings. Firstly, Boyd et al. [17] presented results that demonstrated a significant reduction in AHI following MMA surgery; however, the generalizability of these findings might be restricted by the sample size, the lack of a control group, and potential selection bias, as only patients who underwent MMA were included. Additionally, the retrospective nature of the study could have introduced recall bias or incomplete data capture.

Ceylan et al. [18] compared TCRFTVR and CPAP without finding significant differences, yet this may be due to the limited sensitivity of the outcome measures used to detect clinically meaningful changes or the potential equivalence in efficacy might reflect a type II error due to an underpowered study design. Christino et al. [19] reported enhanced outcomes with additional CCWROP in MMA surgery, but these findings could be influenced by the surgical technique variability and the subjective evaluation of the occlusal plane rotation, leading to potential confounding factors. Cohen et al. [20] indicated a preference for postoperative facial profiles and a reduction in AHI, but the study might suffer from a lack of objective assessment of facial profile changes and a potential bias in patient‐reported outcomes.

Rubio‐Bueno et al. [21] found maxillofacial opening pattern normalisation and mandibular advancement as predictors of treatment success, yet the study's predictive analysis may be limited by the absence of a prospective design and the potential for overfitting in the statistical model used. Weaver et al. [22] concluded that UPPP offered a survival advantage over CPAP; however, the study could be limited by confounders such as patient comorbidities and adherence to CPAP therapy, which were not fully addressed. Woodson et al. [23] found no significant differences between TCRFTA and CPAP, which might be a result of the small sample size or short follow‐up periods that could overlook long‐term outcomes and complications. Yavari et al. [24] observed an initial increase in SBQ scores post‐surgery that later decreased, yet the observational nature of the study without a randomised control might affect the strength of the causal inferences drawn from these results.

### 4.2 Recommendations for Clinical Practice

On the basis of the synthesised information, the following recommendations can be derived for the treatment of OSAS:

1. *MMA surgery*: Given the substantial reduction in AHI scores post‐MMA, with many patients achieving an AHI of less than 5 events/h, MMA surgery should be considered a primary surgical intervention for patients with severe OSA, especially when conservative treatments such as CPAP have failed or are not tolerated. The additional improvements in diastolic BP, ESS scores and FOSQ scores further substantiate the recommendation of MMA as a comprehensive treatment that addresses both the apneic events and the associated cardiovascular and quality of life impairments.
2. *Surgical techniques enhancement*: The enhanced outcomes associated with MMA surgery when combined with CCWROP suggest that incorporating additional occlusal plane rotation into the surgical plan may offer greater benefits in terms of reducing AHI and increasing airway volume. Therefore, surgical plans should be tailored to include such techniques when deemed appropriate based on individual patient anatomies and disease characteristics.
3. *Comparative effectiveness of TCRFTVR and CPAP*: Considering the comparable effectiveness of TCRFTVR and CPAP in improving OSA symptoms, patient preference, tolerance and potential contraindications should be considered when selecting between these options.
4. *UPPP as a treatment alternative*: UPPP should be presented as an alternative treatment option to CPAP, especially in cases where CPAP is not well‐tolerated or adhered to, given the observed survival advantage of UPPP.
5. *Consideration of facial morphology and patient preferences*: The positive reception of postoperative facial profiles after MMA surgery should be taken into account when discussing treatment options with patients, as aesthetic outcomes may influence patient satisfaction and treatment adherence.
6. *Predictors of treatment success*: The normalisation of maxillofacial opening patterns and the extent of mandibular advancement have been identified as predictors of successful treatment outcomes. These factors should be carefully assessed and considered during surgical planning to optimise the chances of treatment success.
7. *Cautious approach to mandibular setback surgery*: Isolated mandibular setback surgery should be approached with caution, as it may increase the risk of OSA, particularly with setbacks greater than 5 mm. Thorough preoperative assessment and postoperative monitoring are recommended to manage and mitigate potential exacerbations in airway obstruction.
8. *Personalised medicine*: The significant heterogeneity observed in the outcomes of various studies highlights the necessity for a personalised medicine approach in the surgical management of OSA. This approach should incorporate individual patient data, disease severity, anatomical characteristics, and comorbidities to inform the selection and customization of surgical interventions.
9. *Clinical trials and further research*: The findings suggest a need for well‐designed, randomised controlled trials with larger sample sizes to further investigate the comparative effectiveness of different surgical interventions and to confirm the predictive factors of treatment success. This research will be paramount in refining patient selection criteria and optimising surgical techniques, with the ultimate goal of enhancing patient outcomes in OSA treatment.

## 5 Conclusion

Our review synthesised data from multiple investigations to evaluate the efficacy of various surgical treatments for OSA. The preponderance of evidence indicated that MMA surgery was effective in substantially reducing the AHI, which marks a significant clinical improvement. This reduction in AHI was accompanied by improvements in diastolic BP and quality of life indicators, such as the ESS and the FOSQ, suggesting that MMA surgery may confer multiple health benefits for patients with OSA. Comparative analyses of therapeutic interventions revealed no significant differences in the effectiveness of TCRFTVR and CPAP therapy, indicating that both modalities may be viable for symptom improvement in OSA patients. Furthermore, adjunctive procedures, such as CCWROP and MMA, were found to enhance airway volumes and further reduce AHI, highlighting the potential value of tailored surgical approaches. The study also noted that specific surgical interventions, such as UPPP, could offer a survival benefit compared to CPAP, although this finding warrants cautious interpretation due to potential confounding factors. Additionally, while TCRFTA and CPAP both improved QOL, no significant differences were observed between these treatments, suggesting that patient preference and individual medical profiles should inform treatment choice.

## Author Contributions

Syed Akbar Ali – data acquisition, manuscript drafting. Maria Maddalena Marrapodi – Study conception, design, data interpretation, manuscript drafting. Ganiga Channaiah Shivakumar – Data extraction and analysis. Sahana Shivakumar – Data extraction and analysis. Jyothikiran Hurkadle – Data interpretation, critical revision. Marco Cicciù – Critical revision. Giuseppe Minervini – Data interpretation, critical revision.

## Ethics Statement

As this study is a systematic review and meta‐analysis, it did not involve direct contact with human participants and therefore did not require ethical approval.

## Conflicts of Interest

The authors declare no conflicts of interest.

### Peer Review

The peer review history for this article is available at https://www.webofscience.com/api/gateway/wos/peer‐review/10.1111/joor.13936.

## Data Availability

The data supporting the findings of this study are available within the article and its Supporting Information.
